# 2-{3,4-Dibut­oxy-5-[5-(3-methyl­phen­yl)-1,3,4-oxadiazol-2-yl]thio­phen-2-yl}-5-(3-methyl­phen­yl)-1,3,4-oxadiazole

**DOI:** 10.1107/S1600536809006539

**Published:** 2009-02-28

**Authors:** Lu-Na Han, Ran-Zhe Lu, Min Zhang, Hai-bo Wang

**Affiliations:** a College of Science, Nanjing University of Technology, Xinmofan Road No. 5 Nanjing, Nanjing 210009, People’s Republic of China; b Department of Applied Chemistry, College of Science, Nanjing University of Technology, Xinmofan Road No. 5 Nanjing, Nanjing 210009, People’s Republic of China

## Abstract

In the title compound, C_30_H_32_N_4_O_4_S, the dihedral angles between the central thio­phene ring and the pendant oxadiazole rings are 10.1 (2) and 6.8 (3)°. The dihedral angles between each oxadiazole ring and its adjacent benzene ring are 6.8 (2) and 5.3 (3)°.

## Related literature

For background on the applications of thio­phenes, see: Laurent *et al.* (2005 ▶).
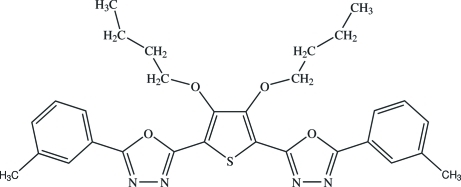


## Experimental

### 

#### Crystal data

C_30_H_32_N_4_O_4_S
*M* *_r_* = 544.67
Monoclinic, 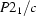

*a* = 16.421 (3) Å
*b* = 14.432 (3) Å
*c* = 12.338 (3) Å
β = 98.36 (3)°
*V* = 2892.9 (11) Å^3^
*Z* = 4
Mo *K*α radiation
μ = 0.15 mm^−1^
*T* = 293 K
0.30 × 0.20 × 0.10 mm

#### Data collection

Enraf–Nonius CAD-4 diffractometer
Absorption correction: ψ scan (North *et al.*, 1968 ▶) *T*_min_ = 0.956, *T*_max_ = 0.985
5526 measured reflections
5260 independent reflections
2161 reflections with *I* > 2σ(*I*)
*R*_int_ = 0.048
3 standard reflections every 200 reflections intensity decay: 1%

#### Refinement

*R*[*F* ^2^ > 2σ(*F* ^2^)] = 0.071
*wR*(*F* ^2^) = 0.167
*S* = 1.00
5260 reflections
352 parameters
52 restraints
H-atom parameters constrained
Δρ_max_ = 0.15 e Å^−3^
Δρ_min_ = −0.13 e Å^−3^

### 

Data collection: *CAD-4 Software* (Enraf–Nonius, 1989 ▶); cell refinement: *CAD-4 Software*; data reduction: *XCAD4* (Harms & Wocadlo, 1995 ▶); program(s) used to solve structure: *SHELXS97* (Sheldrick, 2008 ▶); program(s) used to refine structure: *SHELXL97* (Sheldrick, 2008 ▶); molecular graphics: *SHELXTL* (Sheldrick, 2008 ▶); software used to prepare material for publication: *SHELXL97*.

## Supplementary Material

Crystal structure: contains datablocks global, I. DOI: 10.1107/S1600536809006539/hb2914sup1.cif

Structure factors: contains datablocks I. DOI: 10.1107/S1600536809006539/hb2914Isup2.hkl

Additional supplementary materials:  crystallographic information; 3D view; checkCIF report

## Figures and Tables

**Fig. 1.**
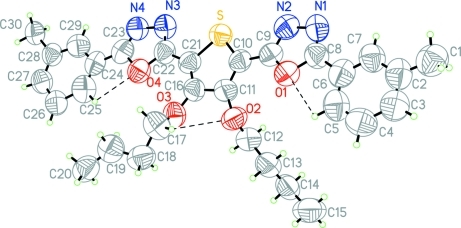
The molecular structure of (I) with displacement ellipsoids for the non-hydrogen atoms drawn at the 30% probability level. Short H···O contacts are shown as dashed lines.

## supplementary crystallographic information

### Comment

Some thiophene derivatives possess biological properties (Laurent *et al.*, 2005). As part of our studies in this area, we report here the synthesis and crystal structure of the title compound, (I).  The molecular structure of (I) is shown in Fig. 1.

### Experimental

3,4-Dibutoxythiophene-2,5-dicarbohydrazide (10 mmol) was dissolved in pyridine (30 ml), and 4-methylbenzoyl chloride (22 mmol) was dropped into the mixture. The resulting mixture was reaction at 348 K for 5 h. After cooling, the mixture was poured into cold water. After filtrating and drying, a white solid compound was obtained.

The crude compound was dissolved in phosphoryl trichloride (30 ml). The mixture was refluxed for 12 h. After cooling, the mixture was poured onto crushed ice. The title compound was obtained and purified by recrystalization from trichloromethane. Yield is 78% and melting point is 440 K. Light yellow blocks of (I) were obtained by slow evaporation of ethyl acetate solution.

### Refinement

All H atoms were placed geometrically (C—H = 0.93–0.96 Å) and refined as riding with *U*_iso_(H) = 1.2*U*_eq_(carrier) or 1.5*U*_eq_(methyl carrier).

### Crystal data

**Table:**

C_30_H_32_N_4_O_4_S	*F*(000) = 1152
*M**_r_* = 544.67	*D*_x_ = 1.251 Mg m^−^^3^
Monoclinic, *P*2_1_/*c*	Melting point: 440 K
Hall symbol: -P 2ybc	Mo *K*α radiation, λ = 0.71073 Å
*a* = 16.421 (3) Å	Cell parameters from 25 reflections
*b* = 14.432 (3) Å	θ = 8–12°
*c* = 12.338 (3) Å	µ = 0.15 mm^−^^1^
β = 98.36 (3)°	*T* = 293 K
*V* = 2892.9 (11) Å^3^	Block, light yellow
*Z* = 4	0.30 × 0.20 × 0.10 mm

### Data collection

**Table:**

Enraf–Nonius CAD-4 diffractometer	2161 reflections with *I* > 2σ(*I*)
Radiation source: fine-focus sealed tube	*R*_int_ = 0.048
graphite	θ_max_ = 25.3°, θ_min_ = 1.3°
ω/2θ scans	*h* = 0→19
Absorption correction: ψ scan (North *et al.*, 1968)	*k* = 0→17
*T*_min_ = 0.956, *T*_max_ = 0.985	*l* = −14→14
5526 measured reflections	3 standard reflections every 200 reflections
5260 independent reflections	intensity decay: 1%

### Refinement

**Table:**

Refinement on *F*^2^	Primary atom site location: structure-invariant direct methods
Least-squares matrix: full	Secondary atom site location: difference Fourier map
*R*[*F*^2^ > 2σ(*F*^2^)] = 0.071	Hydrogen site location: inferred from neighbouring sites
*wR*(*F*^2^) = 0.167	H-atom parameters constrained
*S* = 1.00	*w* = 1/[σ^2^(*F*_o_^2^) + (0.04*P*)^2^ + 1.2*P*] where *P* = (*F*_o_^2^ + 2*F*_c_^2^)/3
5260 reflections	(Δ/σ)_max_ < 0.001
352 parameters	Δρ_max_ = 0.14 e Å^−^^3^
52 restraints	Δρ_min_ = −0.13 e Å^−^^3^

### Special details

**Table:**

Geometry. All e.s.d.'s (except the e.s.d. in the dihedral angle between two l.s. planes) are estimated using the full covariance matrix. The cell e.s.d.'s are taken into account individually in the estimation of e.s.d.'s in distances, angles and torsion angles; correlations between e.s.d.'s in cell parameters are only used when they are defined by crystal symmetry. An approximate (isotropic) treatment of cell e.s.d.'s is used for estimating e.s.d.'s involving l.s. planes.
Refinement. Refinement of *F*^2^ against ALL reflections. The weighted *R*-factor *wR* and goodness of fit *S* are based on *F*^2^, conventional *R*-factors *R* are based on *F*, with *F* set to zero for negative *F*^2^. The threshold expression of *F*^2^ > σ(*F*^2^) is used only for calculating *R*-factors(gt) *etc*. and is not relevant to the choice of reflections for refinement. *R*-factors based on *F*^2^ are statistically about twice as large as those based on *F*, and *R*- factors based on ALL data will be even larger.

### Fractional atomic coordinates and isotropic or equivalent isotropic displacement parameters (Å^2^)

**Table:**

	*x*	*y*	*z*	*U*_iso_*/*U*_eq_	
S	0.26175 (7)	0.22411 (9)	0.70173 (9)	0.0957 (4)	
N1	0.4127 (2)	0.2826 (3)	1.0254 (3)	0.1009 (12)	
O1	0.36204 (17)	0.4067 (2)	0.9317 (3)	0.1033 (10)	
C1	0.5391 (3)	0.4136 (3)	1.4095 (3)	0.1134 (16)	
H1B	0.5566	0.4622	1.4607	0.170*	
H1C	0.5854	0.3751	1.4004	0.170*	
H1D	0.4977	0.3768	1.4366	0.170*	
O2	0.28937 (19)	0.4885 (3)	0.7289 (2)	0.1031 (10)	
N2	0.3700 (2)	0.2498 (3)	0.9236 (3)	0.1017 (12)	
C2	0.5029 (3)	0.4566 (4)	1.2971 (4)	0.0997 (15)	
O3	0.1942 (2)	0.4382 (2)	0.5174 (2)	0.1087 (10)	
N3	0.1668 (3)	0.1439 (4)	0.4892 (3)	0.1150 (14)	
C3	0.5001 (3)	0.5472 (5)	1.2837 (4)	0.1192 (18)	
H3B	0.5219	0.5845	1.3423	0.143*	
O4	0.12985 (18)	0.2718 (2)	0.4131 (3)	0.1015 (9)	
C4	0.4659 (3)	0.5907 (4)	1.1849 (4)	0.1109 (16)	
H4A	0.4644	0.6543	1.1722	0.133*	
N4	0.1195 (3)	0.1222 (3)	0.3878 (4)	0.1079 (13)	
C5	0.4329 (3)	0.5193 (5)	1.1053 (5)	0.122 (2)	
H5A	0.4060	0.5421	1.0391	0.147*	
C6	0.4354 (3)	0.4324 (5)	1.1124 (4)	0.1071 (17)	
C7	0.4737 (3)	0.4002 (4)	1.2129 (4)	0.1107 (16)	
H7A	0.4796	0.3366	1.2228	0.133*	
C8	0.4034 (3)	0.3681 (5)	1.0312 (4)	0.1074 (16)	
C9	0.3451 (3)	0.3246 (4)	0.8750 (4)	0.1014 (15)	
C10	0.2980 (3)	0.3296 (4)	0.7657 (4)	0.0948 (13)	
C11	0.2710 (3)	0.4006 (4)	0.7014 (4)	0.0964 (13)	
C12	0.2256 (3)	0.5477 (4)	0.7441 (4)	0.1291 (18)	
H12A	0.2030	0.5311	0.8099	0.155*	
H12B	0.1820	0.5445	0.6821	0.155*	
C13	0.2629 (3)	0.6454 (4)	0.7548 (5)	0.1299 (19)	
H13A	0.3086	0.6504	0.7134	0.156*	
H13B	0.2219	0.6915	0.7281	0.156*	
C14	0.2906 (3)	0.6583 (4)	0.8693 (5)	0.133 (2)	
H14A	0.3325	0.6114	0.8882	0.160*	
H14B	0.2444	0.6398	0.9054	0.160*	
C15	0.3226 (3)	0.7401 (4)	0.9256 (4)	0.1344 (19)	
H15A	0.3366	0.7269	1.0024	0.202*	
H15B	0.2818	0.7882	0.9156	0.202*	
H15C	0.3709	0.7602	0.8967	0.202*	
C16	0.2334 (3)	0.3697 (4)	0.5944 (4)	0.1108 (16)	
C17	0.2452 (3)	0.4653 (4)	0.4567 (4)	0.1100 (15)	
H17A	0.2766	0.4122	0.4378	0.132*	
H17B	0.2834	0.5080	0.4980	0.132*	
C18	0.2077 (3)	0.5116 (4)	0.3538 (4)	0.1224 (17)	
H18A	0.1648	0.4709	0.3184	0.147*	
H18B	0.1807	0.5672	0.3745	0.147*	
C19	0.2573 (3)	0.5376 (4)	0.2730 (4)	0.1191 (17)	
H19A	0.2880	0.4842	0.2534	0.143*	
H19B	0.2964	0.5847	0.3027	0.143*	
C20	0.2018 (4)	0.5762 (4)	0.1680 (5)	0.161 (2)	
H20A	0.2358	0.5920	0.1137	0.242*	
H20B	0.1732	0.6305	0.1871	0.242*	
H20C	0.1626	0.5298	0.1393	0.242*	
C21	0.2166 (3)	0.2781 (4)	0.5906 (3)	0.0928 (12)	
C22	0.1734 (3)	0.2329 (4)	0.4973 (4)	0.0921 (13)	
C23	0.0989 (3)	0.1984 (5)	0.3461 (5)	0.1063 (16)	
C24	0.0474 (3)	0.2255 (6)	0.2409 (5)	0.1150 (17)	
C25	0.0259 (3)	0.3153 (5)	0.2097 (5)	0.1220 (19)	
H25A	0.0438	0.3633	0.2577	0.146*	
C26	−0.0201 (3)	0.3368 (4)	0.1127 (6)	0.1280 (19)	
H26A	−0.0336	0.3980	0.0948	0.154*	
C27	−0.0465 (4)	0.2655 (6)	0.0408 (5)	0.130 (2)	
H27A	−0.0788	0.2768	−0.0263	0.156*	
C28	−0.0232 (4)	0.1798 (6)	0.0728 (5)	0.126 (2)	
C29	0.0204 (3)	0.1594 (4)	0.1728 (5)	0.1141 (17)	
H29A	0.0310	0.0979	0.1926	0.137*	
C30	−0.0652 (4)	0.1206 (4)	−0.0041 (5)	0.150 (2)	
H30A	−0.0751	0.0645	0.0335	0.225*	
H30B	−0.0290	0.1080	−0.0568	0.225*	
H30C	−0.1164	0.1446	−0.0409	0.225*	

### Atomic displacement parameters (Å^2^)

**Table:**

	*U*^11^	*U*^22^	*U*^33^	*U*^12^	*U*^13^	*U*^23^
S	0.0992 (8)	0.0908 (9)	0.0966 (8)	−0.0007 (7)	0.0129 (6)	0.0017 (7)
N1	0.094 (3)	0.108 (3)	0.098 (3)	0.001 (3)	0.006 (2)	−0.003 (3)
O1	0.100 (2)	0.117 (3)	0.090 (2)	0.0008 (19)	0.0034 (16)	0.002 (2)
C1	0.130 (4)	0.115 (4)	0.093 (3)	−0.009 (3)	0.014 (3)	−0.019 (3)
O2	0.099 (2)	0.106 (3)	0.106 (2)	0.014 (2)	0.0213 (16)	−0.011 (2)
N2	0.096 (3)	0.111 (4)	0.094 (3)	0.017 (2)	0.001 (2)	−0.004 (2)
C2	0.099 (3)	0.113 (4)	0.085 (3)	−0.011 (3)	0.007 (2)	−0.011 (3)
O3	0.123 (2)	0.107 (3)	0.097 (2)	0.000 (2)	0.0192 (18)	0.020 (2)
N3	0.129 (4)	0.115 (4)	0.099 (3)	0.005 (3)	0.010 (2)	0.013 (3)
C3	0.140 (4)	0.122 (5)	0.089 (3)	−0.032 (4)	−0.007 (3)	−0.022 (3)
O4	0.095 (2)	0.108 (3)	0.101 (2)	0.004 (2)	0.0117 (16)	0.006 (2)
C4	0.098 (3)	0.121 (5)	0.115 (4)	−0.014 (3)	0.023 (3)	−0.017 (4)
N4	0.107 (3)	0.117 (4)	0.099 (3)	−0.004 (3)	0.014 (2)	−0.016 (3)
C5	0.097 (4)	0.149 (6)	0.116 (4)	0.007 (4)	−0.004 (3)	−0.010 (5)
C6	0.112 (4)	0.131 (5)	0.079 (3)	0.000 (4)	0.014 (3)	−0.001 (4)
C7	0.097 (3)	0.118 (5)	0.118 (4)	−0.008 (3)	0.020 (3)	−0.003 (4)
C8	0.100 (4)	0.131 (5)	0.091 (3)	0.006 (4)	0.013 (3)	0.005 (4)
C9	0.101 (3)	0.115 (5)	0.086 (3)	−0.011 (3)	0.002 (2)	−0.008 (3)
C10	0.099 (3)	0.098 (4)	0.085 (3)	−0.001 (3)	0.006 (2)	0.008 (3)
C11	0.100 (3)	0.096 (4)	0.097 (3)	−0.005 (3)	0.029 (3)	−0.010 (3)
C12	0.117 (4)	0.122 (5)	0.145 (4)	0.005 (4)	0.007 (3)	−0.014 (4)
C13	0.136 (5)	0.124 (5)	0.135 (5)	0.018 (4)	0.036 (4)	0.026 (4)
C14	0.126 (4)	0.142 (5)	0.132 (4)	0.013 (4)	0.020 (3)	0.000 (4)
C15	0.138 (4)	0.146 (5)	0.126 (4)	0.026 (4)	0.041 (3)	0.022 (4)
C16	0.145 (5)	0.090 (4)	0.093 (3)	0.002 (4)	0.004 (3)	0.007 (3)
C17	0.122 (4)	0.100 (4)	0.109 (3)	−0.001 (3)	0.017 (3)	0.009 (3)
C18	0.146 (4)	0.098 (4)	0.128 (4)	0.001 (3)	0.036 (3)	−0.005 (3)
C19	0.142 (5)	0.092 (4)	0.128 (4)	−0.005 (3)	0.035 (4)	−0.021 (3)
C20	0.192 (6)	0.118 (5)	0.169 (6)	−0.007 (4)	0.010 (5)	0.037 (4)
C21	0.102 (3)	0.094 (3)	0.080 (3)	0.010 (3)	0.005 (2)	0.008 (3)
C22	0.083 (3)	0.102 (4)	0.090 (3)	0.002 (3)	0.009 (2)	0.009 (3)
C23	0.107 (4)	0.107 (5)	0.105 (4)	−0.014 (4)	0.015 (3)	−0.010 (4)
C24	0.098 (4)	0.144 (6)	0.104 (4)	−0.010 (4)	0.015 (3)	0.001 (4)
C25	0.106 (4)	0.142 (6)	0.118 (4)	−0.001 (4)	0.016 (3)	−0.021 (4)
C26	0.113 (4)	0.118 (5)	0.154 (5)	−0.006 (4)	0.023 (4)	0.004 (5)
C27	0.117 (4)	0.148 (6)	0.125 (5)	−0.013 (5)	0.020 (4)	−0.006 (5)
C28	0.115 (5)	0.150 (7)	0.115 (5)	−0.010 (5)	0.021 (4)	−0.018 (5)
C29	0.102 (4)	0.123 (5)	0.115 (4)	−0.010 (3)	0.008 (3)	−0.011 (4)
C30	0.142 (5)	0.169 (6)	0.143 (5)	−0.009 (5)	0.035 (4)	0.007 (5)

### Geometric parameters (Å, °)

**Table:**

S—C21	1.655 (4)	C13—H13A	0.9700
S—C10	1.777 (5)	C13—H13B	0.9700
N1—C8	1.246 (6)	C14—C15	1.432 (6)
N1—N2	1.427 (5)	C14—H14A	0.9700
O1—C9	1.384 (5)	C14—H14B	0.9700
O1—C8	1.427 (5)	C15—H15A	0.9600
C1—C2	1.556 (6)	C15—H15B	0.9600
C1—H1B	0.9600	C15—H15C	0.9600
C1—H1C	0.9600	C16—C21	1.350 (6)
C1—H1D	0.9600	C17—C18	1.486 (6)
O2—C11	1.337 (5)	C17—H17A	0.9700
O2—C12	1.385 (5)	C17—H17B	0.9700
N2—C9	1.274 (6)	C18—C19	1.426 (6)
C2—C3	1.318 (6)	C18—H18A	0.9700
C2—C7	1.352 (6)	C18—H18B	0.9700
O3—C17	1.264 (4)	C19—C20	1.574 (6)
O3—C16	1.456 (5)	C19—H19A	0.9700
N3—C22	1.292 (5)	C19—H19B	0.9700
N3—N4	1.409 (5)	C20—H20A	0.9600
C3—C4	1.413 (6)	C20—H20B	0.9600
C3—H3B	0.9300	C20—H20C	0.9600
O4—C22	1.300 (5)	C21—C22	1.420 (6)
O4—C23	1.394 (6)	C23—C24	1.496 (7)
C4—C5	1.472 (7)	C24—C29	1.306 (7)
C4—H4A	0.9300	C24—C25	1.383 (7)
N4—C23	1.240 (6)	C25—C26	1.355 (7)
C5—C6	1.257 (7)	C25—H25A	0.9300
C5—H5A	0.9300	C26—C27	1.386 (7)
C6—C7	1.387 (6)	C26—H26A	0.9300
C6—C8	1.410 (7)	C27—C28	1.338 (8)
C7—H7A	0.9300	C27—H27A	0.9300
C9—C10	1.456 (6)	C28—C29	1.366 (7)
C10—C11	1.332 (6)	C28—C30	1.383 (7)
C11—C16	1.444 (6)	C29—H29A	0.9300
C12—C13	1.536 (6)	C30—H30A	0.9600
C12—H12A	0.9700	C30—H30B	0.9600
C12—H12B	0.9700	C30—H30C	0.9600
C13—C14	1.431 (6)		
			
C21—S—C10	92.6 (3)	H15A—C15—H15B	109.5
C8—N1—N2	109.3 (5)	C14—C15—H15C	109.5
C9—O1—C8	97.8 (4)	H15A—C15—H15C	109.5
C2—C1—H1B	109.5	H15B—C15—H15C	109.5
C2—C1—H1C	109.5	C21—C16—C11	113.1 (5)
H1B—C1—H1C	109.5	C21—C16—O3	125.0 (5)
C2—C1—H1D	109.5	C11—C16—O3	118.5 (5)
H1B—C1—H1D	109.5	O3—C17—C18	114.7 (4)
H1C—C1—H1D	109.5	O3—C17—H17A	108.6
C11—O2—C12	118.1 (4)	C18—C17—H17A	108.6
C9—N2—N1	102.5 (4)	O3—C17—H17B	108.6
C3—C2—C7	119.9 (5)	C18—C17—H17B	108.6
C3—C2—C1	120.7 (5)	H17A—C17—H17B	107.6
C7—C2—C1	119.4 (5)	C19—C18—C17	120.5 (5)
C17—O3—C16	109.2 (4)	C19—C18—H18A	107.2
C22—N3—N4	108.6 (4)	C17—C18—H18A	107.2
C2—C3—C4	123.5 (5)	C19—C18—H18B	107.2
C2—C3—H3B	118.3	C17—C18—H18B	107.2
C4—C3—H3B	118.3	H18A—C18—H18B	106.8
C22—O4—C23	104.8 (4)	C18—C19—C20	110.3 (5)
C3—C4—C5	109.0 (5)	C18—C19—H19A	109.6
C3—C4—H4A	125.5	C20—C19—H19A	109.6
C5—C4—H4A	125.5	C18—C19—H19B	109.6
C23—N4—N3	104.7 (5)	C20—C19—H19B	109.6
C6—C5—C4	130.3 (6)	H19A—C19—H19B	108.1
C6—C5—H5A	114.8	C19—C20—H20A	109.5
C4—C5—H5A	114.8	C19—C20—H20B	109.5
C5—C6—C7	113.7 (6)	H20A—C20—H20B	109.5
C5—C6—C8	127.1 (6)	C19—C20—H20C	109.5
C7—C6—C8	119.2 (6)	H20A—C20—H20C	109.5
C2—C7—C6	123.4 (6)	H20B—C20—H20C	109.5
C2—C7—H7A	118.3	C16—C21—C22	123.6 (5)
C6—C7—H7A	118.3	C16—C21—S	111.6 (4)
N1—C8—C6	131.1 (6)	C22—C21—S	124.5 (4)
N1—C8—O1	112.6 (5)	N3—C22—O4	109.7 (5)
C6—C8—O1	115.8 (6)	N3—C22—C21	123.1 (5)
N2—C9—O1	117.4 (4)	O4—C22—C21	127.0 (5)
N2—C9—C10	124.7 (5)	N4—C23—O4	112.0 (5)
O1—C9—C10	117.9 (5)	N4—C23—C24	132.7 (6)
C11—C10—C9	132.5 (5)	O4—C23—C24	115.3 (6)
C11—C10—S	109.5 (4)	C29—C24—C25	117.2 (6)
C9—C10—S	117.9 (4)	C29—C24—C23	117.6 (7)
C10—C11—O2	122.4 (5)	C25—C24—C23	125.2 (6)
C10—C11—C16	111.6 (5)	C26—C25—C24	123.1 (6)
O2—C11—C16	125.2 (5)	C26—C25—H25A	118.4
O2—C12—C13	106.1 (5)	C24—C25—H25A	118.4
O2—C12—H12A	110.5	C25—C26—C27	118.6 (7)
C13—C12—H12A	110.5	C25—C26—H26A	120.7
O2—C12—H12B	110.5	C27—C26—H26A	120.7
C13—C12—H12B	110.5	C28—C27—C26	116.6 (7)
H12A—C12—H12B	108.7	C28—C27—H27A	121.7
C14—C13—C12	105.6 (5)	C26—C27—H27A	121.7
C14—C13—H13A	110.6	C27—C28—C29	123.7 (7)
C12—C13—H13A	110.6	C27—C28—C30	106.0 (7)
C14—C13—H13B	110.6	C29—C28—C30	129.1 (8)
C12—C13—H13B	110.6	C24—C29—C28	120.5 (7)
H13A—C13—H13B	108.7	C24—C29—H29A	119.7
C13—C14—C15	128.9 (6)	C28—C29—H29A	119.7
C13—C14—H14A	105.1	C28—C30—H30A	107.0
C15—C14—H14A	105.1	C28—C30—H30B	106.6
C13—C14—H14B	105.1	H30A—C30—H30B	109.5
C15—C14—H14B	105.1	C28—C30—H30C	114.7
H14A—C14—H14B	105.9	H30A—C30—H30C	109.5
C14—C15—H15A	109.5	H30B—C30—H30C	109.5
C14—C15—H15B	109.5		
			
C8—N1—N2—C9	5.5 (6)	O2—C11—C16—C21	−176.0 (4)
C7—C2—C3—C4	1.6 (9)	C10—C11—C16—O3	174.7 (4)
C1—C2—C3—C4	−178.1 (4)	O2—C11—C16—O3	−15.8 (7)
C2—C3—C4—C5	1.9 (7)	C17—O3—C16—C21	−108.3 (6)
C22—N3—N4—C23	−2.9 (6)	C17—O3—C16—C11	94.0 (5)
C3—C4—C5—C6	−3.9 (9)	C16—O3—C17—C18	161.7 (4)
C4—C5—C6—C7	1.8 (10)	O3—C17—C18—C19	−174.0 (5)
C4—C5—C6—C8	−178.8 (5)	C17—C18—C19—C20	174.6 (4)
C3—C2—C7—C6	−4.2 (8)	C11—C16—C21—C22	174.4 (4)
C1—C2—C7—C6	175.5 (4)	O3—C16—C21—C22	15.8 (8)
C5—C6—C7—C2	2.5 (8)	C11—C16—C21—S	−12.2 (6)
C8—C6—C7—C2	−177.0 (5)	O3—C16—C21—S	−170.9 (4)
N2—N1—C8—C6	−177.4 (5)	C10—S—C21—C16	5.6 (4)
N2—N1—C8—O1	−6.6 (6)	C10—S—C21—C22	178.9 (4)
C5—C6—C8—N1	168.7 (6)	N4—N3—C22—O4	4.3 (5)
C7—C6—C8—N1	−11.9 (9)	N4—N3—C22—C21	179.2 (4)
C5—C6—C8—O1	−1.8 (9)	C23—O4—C22—N3	−3.9 (5)
C7—C6—C8—O1	177.6 (4)	C23—O4—C22—C21	−178.5 (4)
C9—O1—C8—N1	4.7 (5)	C16—C21—C22—N3	170.8 (5)
C9—O1—C8—C6	177.0 (4)	S—C21—C22—N3	−1.6 (7)
N1—N2—C9—O1	−2.5 (6)	C16—C21—C22—O4	−15.3 (8)
N1—N2—C9—C10	179.4 (4)	S—C21—C22—O4	172.3 (3)
C8—O1—C9—N2	−0.9 (5)	N3—N4—C23—O4	0.4 (6)
C8—O1—C9—C10	177.2 (4)	N3—N4—C23—C24	−178.7 (5)
N2—C9—C10—C11	−176.1 (5)	C22—O4—C23—N4	2.1 (6)
O1—C9—C10—C11	5.9 (8)	C22—O4—C23—C24	−178.6 (4)
N2—C9—C10—S	8.1 (7)	N4—C23—C24—C29	−4.4 (9)
O1—C9—C10—S	−169.9 (3)	O4—C23—C24—C29	176.6 (4)
C21—S—C10—C11	2.7 (4)	N4—C23—C24—C25	175.6 (6)
C21—S—C10—C9	179.4 (4)	O4—C23—C24—C25	−3.4 (8)
C9—C10—C11—O2	4.4 (8)	C29—C24—C25—C26	−0.9 (8)
S—C10—C11—O2	−179.6 (3)	C23—C24—C25—C26	179.1 (5)
C9—C10—C11—C16	174.3 (5)	C24—C25—C26—C27	−0.4 (8)
S—C10—C11—C16	−9.7 (5)	C25—C26—C27—C28	−0.9 (8)
C12—O2—C11—C10	−116.6 (5)	C26—C27—C28—C29	3.6 (9)
C12—O2—C11—C16	74.9 (6)	C26—C27—C28—C30	172.3 (5)
C11—O2—C12—C13	−170.5 (4)	C25—C24—C29—C28	3.5 (8)
O2—C12—C13—C14	−90.1 (5)	C23—C24—C29—C28	−176.5 (5)
C12—C13—C14—C15	−173.2 (6)	C27—C28—C29—C24	−5.2 (9)
C10—C11—C16—C21	14.5 (6)	C30—C28—C29—C24	−171.1 (6)
